# Immunomodulatory Activities of Periodontal Ligament Stem Cells in Orthodontic Forces-Induced Inflammatory Processes: Current Views and Future Perspectives

**DOI:** 10.3389/froh.2022.877348

**Published:** 2022-05-04

**Authors:** Christian Behm, Zhongqi Zhao, Oleh Andrukhov

**Affiliations:** Competence Center Periodontal Research, University Clinic of Dentistry, Medical University of Vienna, Vienna, Austria

**Keywords:** orthodontic tooth movement, non-infectious inflammation, periodontal ligament, periodontal ligament stem cells, immunomodulation

## Abstract

Orthodontic tooth movement (OTM) is induced by applying active mechanical forces, causing a local non-infectious inflammatory response in the periodontal ligament (PDL). As a prerequisite for OTM, the inflammation status is associated with increased levels of various cytokines and involves the interaction between immune cells and periodontal ligament stem cells (hPDLSCs). It is well established that hPDLSCs respond to orthodontic forces in several ways, such as by secreting multiple inflammatory factors. Another essential feature of hPDLSCs is their immunomodulatory activities, which are executed through cytokine (e.g., TNF-α and IL-1β)-induced production of various soluble immunomediators (e.g., indoleamine-2,3-dioxygenase-1, tumor necrosis factor-inducible gene 6 protein, prostaglandin E_2_) and direct cell-to-cell contact (e.g., programmed cell death ligand 1, programmed cell death ligand 2). It is well known that these immunomodulatory abilities are essential for local periodontal tissue homeostasis and regeneration. So far, only a handful of studies provides first hints that hPDLSCs change immunological processes during OTM via their immunomodulatory activities. These studies demonstrate the pro-inflammatory aspect of immunomodulation by hPDLSCs. However, no studies exist which investigate cytokine and cell-to-cell contact mediated immunomodulatory activities of hPDLSCs. In this perspective article, we will discuss the potential role of the immunomodulatory potential of hPDLSCs in establishing and resolving the OTM-associated non-infectious inflammation and hence its potential impact on periodontal tissue homeostasis during OTM.

## Introduction

The skeletal and immune systems are two functionally interconnected organizational units that affect each other by sharing multiple cells and mediators, such as cytokines, receptors and other signaling molecules. A prominent example of this interplay is the orthodontic tooth movement (OTM) [1], in which a complex and highly coordinated non-infectious inflammation of periodontal tissues is an essential pre-requisite [2]. The inflammatory processes are induced by applying orthodontic forces, generating compression and tension areas within the periodontal ligament (PDL) [3, 4]. Blood vessel and nerve endings occlusion and hypoxia trigger periodontal vasodilation and the extravasation of various immune cells [2, 4]. Among other tissue-resident cells, immune cells, such as neutrophiles, macrophages, T and B lymphocytes, may contribute to increased levels of various immune factors in the periodontium, such as various chemokines, growth factors, cytokines [e.g., interleukin-(IL)-1β, IL-6, IL-8, tumor necrosis factor-(TNF)-α, interferon-(IFN)-γ], and prostaglandin E_2_ (PGE_2_). All these inflammatory mediators contribute to the regulation of osteoclast- and osteogenesis and the activities of bone remodeling cells (osteoclasts and osteoblasts) [1, 5]. Hence, the inflammatory environment is directly involved in orchestrating the interconnected catabolic (bone resorption) and anabolic (bone formation) processes within the periodontium, which finally leads to alveolar bone turnover and tooth movement [6]. In 1962, Burstone [7] divided OTM into three different phases, depending on the tooth displacement rate. More recently, this model was updated, proposing four distinct OTM phases (initial phase, lag phase, acceleration phase and linear phase). The initial phase is induced immediately after applying orthodontic forces and lasts between 24 and 48 h. It is characterized by an instantaneous tooth displacement within the alveolar socket. Due to emerging necrosis and hyalinization of the PDL and the adjacent tissue, the initial phase is followed by the lag phase exhibiting low or even no tooth displacement. The lag phase persists for 20–30 days. After necrotic and hyalinized tissue is completely cleared by osteoclasts and phagocytes during a process called undermining resorption, tooth displacement rate starts to increase gradually, turning into a continuous rate of tooth movement during the linear phase (rapid OTM) as long as mechanical stimuli persist [8–10].

Apart from immune cells, it is well established that the cellular component of the PDL functions as primary mechanosensors, translating mechanical stimuli into biological signals [11, 12]. The cells in the PDL are a heterogeneous population with a fibroblast-like morphology containing endothelial, neuronal and epithelial cells, osteoblasts, fibroblasts and mesenchymal stem/stromal cells (MSCs) [13, 14], exhibiting progenitor and self-renewing potentials [14–16]. Plenty of studies on OTM used the whole heterogeneous cell bulk from the PDL, naming them as PDL cells or PDL fibroblasts [5]. There are also studies, terming them periodontal ligament stem cells (hPDLSCs), although comparable isolation methods were used (e.g., [17–22]). Additionally, these studies mainly verify the stemness of isolated cells in accordance with the minimal criteria for MSCs as settled by the International Society for Cell and Gene Therapy (ISCT) [15]: plastic adherence *in vitro*, expression of specific surface markers and tri-lineage differentiation potential *in vitro*. Due to uniformity, we will name the cells isolated from the PDL as hPDLSCs throughout the whole article. As primary mechanosensors within the PDL, hPDLSCs contribute to the force-induced alveolar bone and PDL remodeling and consequently to OTM via multiple different ways (reviewed in Li et al. [5] and Huang et al. [11]). Orthodontic forces change the proliferation and osteogenic differentiation capacity of hPDLSCs. They directly affect osteoclast- and osteogenesis via the production of receptor activator of NF-κB ligand (RANKL) and osteoprotegerin (OPG). hPDLSCs produce multiple other regulatory molecules, establishing a signaling axis between them and osteoblasts, osteoclasts and osteocytes. They also secrete anabolic as well as catabolic molecules, involved in remodeling the extracellular matrix (ECM) of the PDL during OTM [5, 11]. Furthermore, hPDLSCs appears to be the most important player in triggering mechanical forces-induced non-infectious inflammation via the secretion of various pro-inflammatory factors [3, 5, 23, 24]. Beside all these regulatory mechanisms, their well-defined immunomodulatory activities (reviewed in Andrukhov et al. [25]) have been neglected as possible regulators of OTM, so far. Hence, this perspective article will be restricted to the potential role of the cytokine-boosted immunomodulatory properties of hPDLSCs in the context of OTM. The role of other cellular processes involved in OTM, such as chemotaxis, homing and the role of damage-associated patterns will be out the scope of this article and are discussed elsewhere (e.g., [5]).

## Immune Cell Modulation by hPDLSCs in Different OTM Phases

While the role of cytokine secretion by hPDLSCs in inducing non-infectious inflammation during OTM is well established, the involvement of different immune cells during OTM still needs extensive research [5]. Nevertheless, there are obvious hints that innate as well as adaptive immune cells are involved in the progress of aseptic inflammation during OTM, by secreting chemokines, pro- and anti-inflammatory cytokines and by contributing to vasodilation and necrotic tissue removal. However, the precise immunological mechanisms remain vague.

The inflammatory reactions during OTM are hypothesized to be highly dynamic and specific for each OTM phase (reviewed in Chaushu et al. [2]). The initial phase seems to be characterized by acute inflammatory reactions, involving typical innate immune responses from periodontal tissue-resident and extravasated immune cells and leading to high levels of various pro-inflammatory cytokines. During the further course of OTM, the authors claim that the induced inflammation seems to be resolved. However, this resolution appears incomplete, turning into a low-grade chronic inflammation. These low-grade chronic inflammatory processes may persist during the acceleration and linear phases until the force is renewed, which facilitates a new acute inflammatory reaction.

**Figure 1:**
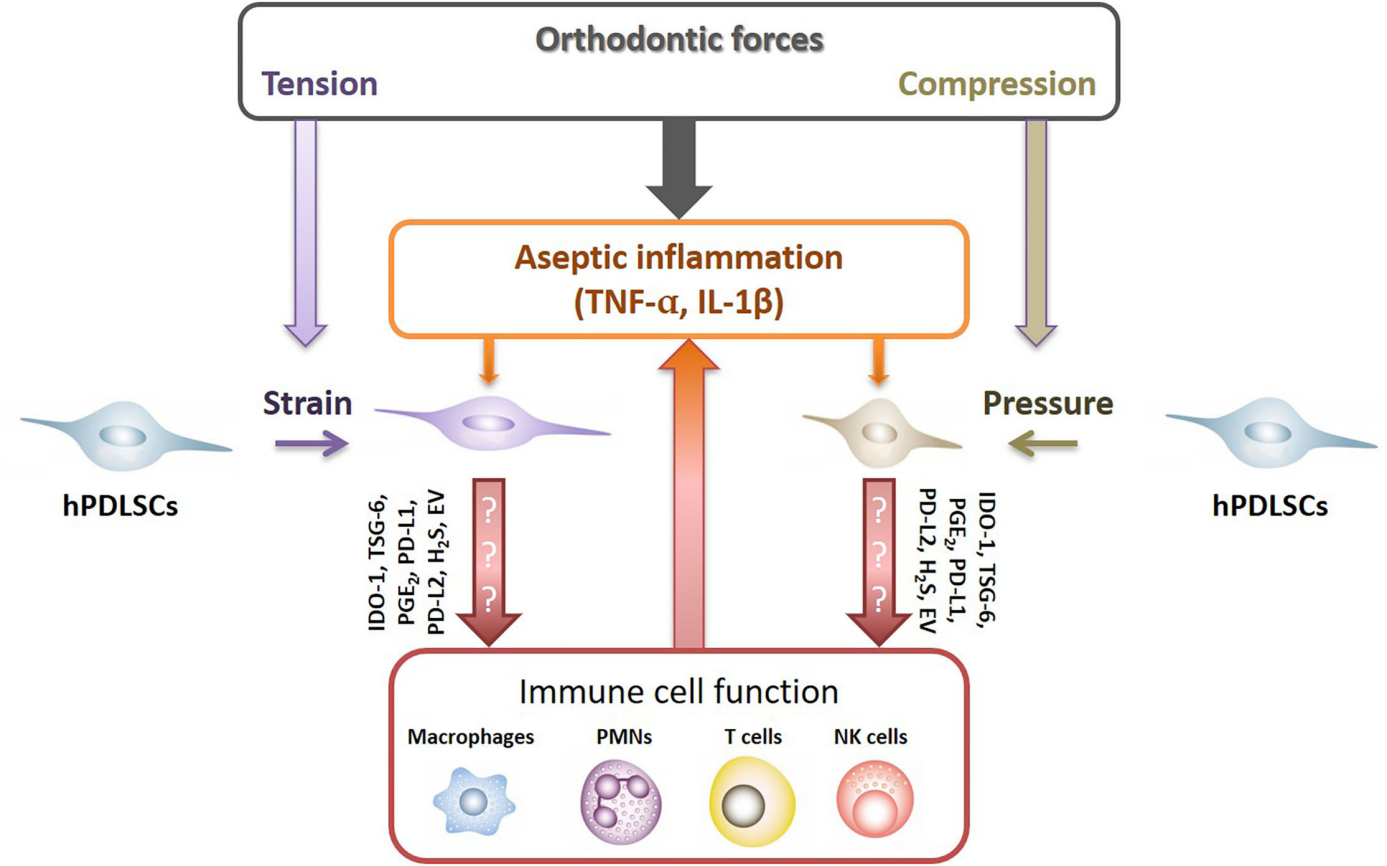
Potential crosstalk between hPDLSCs and immune cells after applying orthodontic forces. Under inflammatory conditions hPDLSCs significantly modulate local immune responses by affecting various immune cells, such as macrophages, neutrophiles (PMNs), NK cells and T lymphocytes. hPDLSCs execute their immunomodulatory activities via various mechanisms which are boosted by various pro-inflammatory cytokines, such as TNF-α, IL-1β, and IFN-γ [25–29]. Hence, it seems likely that the non-infectious inflammatory conditions during OTM modulate the immunomodulatory activities of hPDLSCs, which in turn should influence the activity of immune cells. Indoleamine-2,3-dioxygenase-1 (IDO-1), tumor necrosis factor-inducible gene 6 protein (TSG-6), prostaglandin E_2_ (PGE_2_), programmed cell death ligand 1/2 (PD-L1/2), hydrogen sulfide (H_2_S), extracellular vesicles (EV).

It is well explored that under inflammatory environment hPDLSCs are fundamental modulators of the local immune responses, affecting various immune cells, such as macrophages, granulocytes, dendritic cells, B and T lymphocytes [25]. hPDLSCs execute their immunomodulatory activities via various mechanisms, such as the expression of soluble (e.g., indoleamine-2,3-dioxygenase-1, prostaglandin E_2_, tumor necrosis factor-inducible gene 6 protein) and membrane-bound (e.g., programmed cell death ligand-1/2) immunomediators. Since the immunomediator production get boosted by various cytokines, such as TNF-α, IL-1β, and IFN-γ [25–29], it may be possible that the aseptic inflammatory environment during OTM modulates the immunomodulatory properties of hPDLSCs, which, in turn, should affect immune cells' activity and inflammatory reaction (Figure 1). Additionally, recent studies have also demonstrated that MSCs' secreted extracellular vesicles are involved in the immunomodulatory crosstalk between MSCs and immune cells [30]. However, if and how far the aseptic inflammation may be also affected by these immunomodulatory mechanisms of hPDLSCs remains largely unknown. So far, only few recently published studies gave first clues that orthodontic forces may impact the immunomodulatory abilities of hPDLSCs [31–35].

### Initial Phase

The acute inflammatory reaction in the initial OTM phase is characterized by an increase in infiltrating neutrophils within 3 days after applying orthodontic forces [36–38]. If orthodontic forces may change immunomodulatory activities of hPDLSCs against neutrophils has not been investigated, so far. However, several studies already observed inhibitor effects of hPDLSCs toward the apoptosis of neutrophils in the absence of mechanical forces [39, 40]. Misawa et al. [41] additionally showed decreased production of reactive oxygen species in neutrophils, caused by conditioned medium from hPDLSCs.

Several studies demonstrated enhanced monocyte levels [37, 38] and an increase in M1 pro-inflammatory macrophages [42–44] in the initial OTM phase, which is accompanied by the secretion of various pro-inflammatory cytokines [45]. Three recently published studies showed that force-stimulated hPDLSCs might be involved in triggering macrophages toward a pro-inflammatory phenotype. He et al. [32] observed increased expression of pro-inflammatory cytokines in *in vitro* cultured macrophages after adding conditioned medium from hPDLSCs treated with mechanical forces through hydrogen sulfide (H_2_S) production. Jiang et al. [33] also showed increased M1 macrophage polarization after stimulating macrophages with supernatant of hPDLSCs, which were treated with compressive forces for 12 h *in vitro*. Zhao et al. [46] also demonstrated enhanced *in vitro* M1 macrophage polarization by adding exosomes secreted from hPDLSCs which were stimulated with static compressive forces for 4 h. It should be noted that these three studies [32, 33, 46] were conducted in the absence of any inflammatory stimuli and this might underlie the promotion of M1 polarization by mechanically treated hPDLSCs. Most studies imply that hPDLSCs favor M2 anti-inflammatory phenotype; however, this ability is enhanced by the inflammatory environment and in absence of any mechanical forces [47–50], whereas one study [47] revealed an enhanced polarization to the M1 pro-inflammatory phenotype in the presence of extracellular vesicles derived from lipopolysaccharide-treated hPDLSCs in the absence of mechanical forces. Besides the influence of hPDLSCs on the macrophages' polarization status, Wolf et al. [34] also showed *in vitro* that supernatant from hPDLSCs, treated with compressive forces for 24 h, enhanced macrophage migration and their osteoclast differentiation.

Granulocytes/mast cells also seem to have a role in OTM, at least during the initial stage. Yamasaki et al. [51] observed a significant decrease of mast cells immediately after applying orthodontic forces, suggesting a release of histamines by degranulation. Groeger et al. [52] demonstrated a histamine-induced increase in the expression of pro-inflammatory cytokines and RANKL in hPDLSCs, which might promote osteoclastogenesis. It has still to be elucidated if mechanical forces influence the immunomodulatory effects of hPDLSCs on mast cells. To date, it is also unknown if hPDLSCs and MSCs from other dental tissues generally affect mast cell functions. A few studies showed that non-dental MSCs suppressed mast cell degranulation, cytokine production and chemotaxis [53–55].

Information about remaining innate immune cells, concerning their contribution to OTM as well as their interaction with hPDLSCs, is very scant. So far, only one study observed increased activation of NK cell-associated signaling pathways [38]. The involvement of dendritic cells in OTM was merely demonstrated by Vandevska-Radunovic et al. [42] showing increased levels of CD11^+^ cells after applying mechanical forces. Low levels of γδ T lymphocytes are observed immediately after initiating OTM, but their importance was shown by Wald et al. [37], reporting a significantly reduced tooth displacement after their depleting. So far, there are no studies investigating the modulatory activities of hPDLSCs in the presence of orthodontic forces toward natural killer cells (NK cells), dendritic cells and γδ T lymphocytes. In the absence of orthodontic forces, only one study showed that hPDLSCs affect dendritic cells by suppressing the expression of non-classical major histocompatibility complex-like glycoprotein CD1b [56], whereas no information exists about immunomodulatory activities of hPDLSCs on NK cells and γδ T lymphocytes. MSCs isolated from the gingiva and human exfoliated deciduous teeth suppress the activation and maturation of dendritic cells [57, 58]. Non-dental MSCs were shown to suppress the activity of NK cells [59] and proliferation of γδ T lymphocytes [60, 61].

Beside the innate branch of the immune system, also the adaptive immune system seems to be involved in the initial OTM phase, too [2]. Particularly, Kook et al. [35] observed an increase in CD220^+^ B lymphocytes, which seems to sustain until the linear phase, according to Wald et al. [37]. Nevertheless, the exact role of B lymphocytes is ambiguously, although B lymphocytes are a known potent RANKL source [2]. So far, no study has investigated the influence of hPDLSCs on B lymphocytes when applying orthodontic forces. Only one study examined the immunomodulatory activities of hPDLSCs on B lymphocytes. Lie et al. observed immunosuppressive effects of hPDLSCs on the differentiation, chemotaxis and proliferation of B lymphocytes, basically mediated by a direct cell-to-cell contact [62].

Studies also observed increased CD4^+^ T lymphocyte levels [35] and enhanced number of pro-inflammatory Th17 and Th1 lymphocytes in the initial OTM phase [63]. This increase was verified by Yan et al. [64], demonstrating IFN-γ and TNF-α secretion during the early OTM. On the contrary, Ogawa et al. [65] showed that depleting CD4^+^ or CD8^+^ T lymphocytes caused no reduction in orthodontic tooth displacement. Hence, the role of T lymphocytes in OTM is questionable and needs further research. However, studies already exist which reveal an effect of orthodontic forces-exposed hPDLSCs on T lymphocytes. Kook et al. [35] observed increased TNF-α secretion from hPDLSCs at the compression side, which facilitated RANKL expression in CD4^+^ T lymphocytes. Additionally, Lin et al. [31] declared enhanced Th17 differentiation of CD4^+^ T lymphocytes in the presence of conditioned medium from hPDLSCs treated with heavy compressive forces, mediated by increased IL-6 and decreased TGF-β secretion. Thus, this study demonstrated, for the first time, that hPDLSCs might contribute to the mechanical forces-induced inflammatory status [31], but it should be noted that these experiments were conducted in a non-inflammatory setting. In contrast, in the absence of orthodontic forces and in the presence of IFN-γ hPDLSCs inhibited IL-17 expression of T lymphocytes and stimulated T_regs_ differentiation [66]. This indicates that the presence/absence of inflammatory stimuli/mechanical forces may change the immunomodulatory activities of hPDLSCs on T lymphocytes. Additionally, several studies showed that in the absence of mechanical forces, hPDLSCs inhibit the proliferation of T lymphocytes and peripheral blood mononuclear cells [25, 29, 56, 66–68].

### Lag Phase

Only a few studies have investigated inflammatory processes within the lag phase of OTM. Studies indicated that the percentage of neutrophils, monocytes and inflammatory subtypes of αβ T lymphocytes decreased, whereas that of anti-inflammatory regulatory T lymphocytes increased [37, 38, 63]. To date, no study has examined the influence of mechanically-triggered hPDLSCs on these immune cells during the lag phase of OTM.

One study demonstrated an accumulation of pro-inflammatory M1 macrophages and no changes in the levels of anti-inflammatory M2 macrophages [43]. This accumulation could be due to the promotion of M1 polarization by hPDLSCs, as we already mentioned above. Jiang et al. [33] observed increased number of M1 macrophages at the compression side of the PDL in a rat model after applying orthodontic forces for 7 days. This increase may be induced due to enhanced autophagy of hPDLSCs, induced by applied mechanical forces. In addition, He et al. [32] also observed increased M1 macrophage polarization at the compression side of the PDL after applying orthodontic forces to mice for 7 days, which might be mediated by the hPDLSCs-dependent production of H_2_S.

Despite this first evidence of incompletely dampened inflammatory processes and the role of immunomodulatory activities of hPDLSCs during these processes, the exact inflammatory processes and their modulation by hPDLSCs in this stage have still to be elucidated.

### Acceleration/Linear Phase

Only a few studies explored immunological processes within the acceleration/linear phase. He et al. [43] exhibited a decrease in M1 macrophages. Additionally, a decrease in neutrophils, monocytes, γδ and CD4^+^ T lymphocytes was observed [2, 37]. Interestingly, these levels were still significantly higher compared to the levels before applying orthodontic forces. Further, Wald et al. [37] observed increased levels of B and CD3^+^ lymphocytes. Together this indicates that a low-grade chronic inflammation persists during linear OTM until the force is renewed [2]. Further studies are highly essential to elucidate the exact immunological mechanisms confirming the suggested low-grade chronic inflammation during the linear phase of OTM. To the best of our knowledge, if and how immunomodulatory activities of hPDLSCs are involved in this proposed low-grade chronic inflammation has not been investigated so far. Since the immunomodulatory potential of hPDLSCs is mainly immunosuppressive [25], a contribution to this (partly) resolved inflammation may be possible.

## Hypothetical Regulation of Immune Cells During OTM *via* Immunomodulatory Activities of hPDLSCs

Besides first hints that mechanical force-triggered hPDLSCs influence immune cells during OTM by their immunomodulatory activities [31–35], there are several other facts that allow the assumption that immunomodulatory properties participate in regulating inflammatory processes during OTM. First, several immunomediators, such as IL-10 and TGF-β, which are secreted by hPDLSCs [25], are known to participate in OTM by inducing osteogenesis [69]. Second, the acute inflammatory phase during the initial OTM stage is characterized by a significant increase in various pro-inflammatory factors, such as IL-β, TNF-α, and IFN-γ [1]. These cytokines are also potent activators of paracrine and direct cell-to-cell immunomodulatory mechanisms of hPDLSCs. Moreover, certain cytokines or a combination of cytokines activate only specific immunomodulatory functions [25, 29], which may contribute to the fine-tuning of the aseptic inflammation during OTM.

Moreover, hPDLSCs adopt a pro- or anti-inflammatory phenotype in the presence of low or high cytokine levels, respectively [27]. Hence, we suggest a potential mechanism for how hPDLSCs are involved in regulating the initiation of acute inflammation and its transition to a low-grade chronic inflammation during OTM: (1) Immediately after applying orthodontic forces the combination of mechanical forces and low cytokine levels may facilitate the adaption of a pro-inflammatory phenotype of hPDLSCs with increased secretion of various cytokines [3, 5, 23, 24] and possible reduced activity of immunomodulatory mechanisms (C.B. and O.A., unpublished data). These together may facilitate immune cell recruitment and activation, contributing to the aseptic inflammatory environment during the initial OTM stage. (2) Subsequently, high cytokine level-triggered immunomodulatory activities of hPDLSCs may initiate an anti-inflammatory phenotype, contributing to the resolution of the inflammatory reaction in the further course of OTM. Dampening the cytokine-induced immunomodulatory mechanisms of hPDLSCs through applied orthodontic forces, however, to a level that is still higher compared to unstimulated hPDLSCs (C.B. and O.A., unpublished data), may contribute to the potential partial nature of the inflammatory resolution [2].

## Conclusion and Future Perspective

In conclusion, hPDLSCs are well-established key players in initiating non-infectious inflammation in the PDL during OTM. There are already first hints that orthodontic forces influence immunomodulatory mechanisms of hPDLSCs and hence the interaction between them and various immune cells [31–35]. hPDLSCs are highly potential candidates involved in orchestrating the timely-dynamic aseptic inflammatory reactions during OTM. In this regard, their mainly immunosuppressive immunomodulatory property [25] could be the secret player. It can affect the course of aseptic inflammation (Figure 1) as well as might also indirectly influence bone resorption through the modulation of OPG/RANKL production by immune cells [1]. Unfortunately, only a handful of studies have already examined the direct influence of orthodontic force-triggered hPDLSCs on various immune cells [31–35, 70]. How far immunomodulatory activities of hPDLSCs are involved in the initial stage of OTM and in transforming acute inflammation into a potential low-grade chronic inflammation [2] need further extensive research.

Future *in vivo* studies should investigate the involvement of various MSCs-dependent immunomodulatory mechanisms in OTM by trailing the levels of soluble immunomediators in the gingival crevicular fluid and saliva of OTM patients and by depleting them in different animal models. *In vitro* studies should explore the expression of various immunomediators in hPDLSCs and their effect on various immune cells in the context of OTM. These experiments should be performed using different types of co-culture models and various inflammatory environments. Since the response of hPDLSCs on mechanical forces depends on the force type and amplitude [71, 72], the effect of these factors must be considered.

Deciphering the involvement of hPDLSCs derived immunomodulation in OTM may also benefit orthodontic patients directly. The inflammatory component of OTM has an immense attention as a point of action for the pharmacological acceleration of orthodontic tooth displacement [2]. The immunomodulatory mechanisms of hPDLSCs may also function as a new contact point for accelerating OTM pharmacologically. Furthermore, over the years, the number of adults with orthodontic treatment has substantially increased. In this patient group, the incidence of periodontitis is significantly higher, which might negatively influence orthodontic treatment [73, 74], by triggering a periodontitis-associated dormant inflammation [73, 75]. It is already known that hPDLSCs from periodontitis patients exhibit reduced immunosuppressive activities (reviewed in Andrukhov et al. [25]). Unraveling the behavior of immunomodulatory potential of hPDLSCs during OTM may further shed light on the OTM-periodontitis interaction. Additionally, an undesirable side-effect of applying orthodontic forces is root resorption. Animal studies already demonstrated that MSCs inhibit orthodontically-induced root resorption (OIRR) and/or trigger reparative mechanisms. It is thought that this is achieved by the cementogenic differentiation potential of hPDLSCs [76–78]. If and how their immunomodulatory properties are mechanistically involved in OIRR needs further investigation.

## Data Availability Statement

The original contributions presented in the study are included in the article/supplementary material, further inquiries can be directed to the corresponding author.

## Author Contributions

CB, ZZ, and OA contributed to the design of the work. CB wrote the original draft, which was edited by ZZ and OA. All authors approved the final version of the manuscript.

## Funding

This research was funded by Austrian Science Fund (FWF); Grant Number P 35037 (to OA).

## Conflict of Interest

The authors declare that the research was conducted in the absence of any commercial or financial relationships that could be construed as a potential conflict of interest.

## Publisher's Note

All claims expressed in this article are solely those of the authors and do not necessarily represent those of their affiliated organizations, or those of the publisher, the editors and the reviewers. Any product that may be evaluated in this article, or claim that may be made by its manufacturer, is not guaranteed or endorsed by the publisher.
